# Prediction of Clinical Outcomes With EEG Microstate in Patients With Major Depressive Disorder

**DOI:** 10.3389/fpsyt.2021.695272

**Published:** 2021-08-16

**Authors:** Danfeng Yan, Jin Liu, Mei Liao, Bangshan Liu, Shibin Wu, Xueqin Li, Haolun Li, Wenwen Ou, Li Zhang, Zexuan Li, Yan Zhang, Lingjiang Li

**Affiliations:** ^1^National Clinical Research Center for Mental Disorders, Department of Psychiatry, The Second Xiangya Hospital of Central South University, Changsha, China; ^2^Hunan Key Laboratory of Psychiatry and Mental Health, Institute of Mental Health, Hunan Medical Center of Mental Health, China National Technology Institute on Mental Disorders, Changsha, China; ^3^Shanxi Mental Health Center, Taiyuan, China; ^4^Department of Psychiatry, Nanning Fifth People's Hospital, Nanning, China

**Keywords:** major depressive disorders, drug-naïve patient, electroencephalogram, EEG microstate, first-episode depression

## Abstract

**Background:** The difficulty in timely evaluating patient response to antidepressants has brought great challenge to the treatment of major depressive disorder (MDD). Some studies found that the electroencephalogram (EEG) microstates might be a reliable marker to evaluate patient response to treatment. The present study aims to evaluate the relationship between EEG microstate parameters and MDD symptoms before and after treatment to identify predictive biological markers for patient response.

**Methods:** Thirty drug-naïve MDD patients (20 females and 10 males) were enrolled in this study. All the patients received effective dosages of selective serotonin reuptake inhibitors, and EEG recordings were collected at baseline and 2 weeks of treatment. Brain activities during the eyes-closed state were recorded using 64-channel electroencephalography, and the patients' microstates were clustered into four maps according to their topography (labeled A, B, C, and D). The differences of EEG microstates before and after treatment were compared using paired *t*-test. Spearman correlation coefficients were calculated to identify the relationships between the improvement of depression and anxiety symptoms and microstate parameters.

**Results:** The mean duration (69.67 ± 10.33 vs. 64.00 ± 7.70, *p* < 0.001) and occurrence (4.06 ± 0.69, vs. 3.69 ± 0.70, *p* = 0.002) of microstate B decreased significantly after treatment. The proportion of microstate B also decreased (27.53 ± 5.81, vs. 23.23 ± 4.61, *p* < 0.001), while the occurrence of microstate A increased after treatment. A significant negative correlation was found between the change of score of Hamilton Rating Scale for Anxiety and the increase of the occurrence of microstate A (*r* = −0.431, *p* < 0.05) after 2 weeks of treatment. The reduction of the duration of microstate B was found to be predictive of patient response to antidepressants after 3 months.

**Conclusion:** This study explored the relationship between changes of EEG microstates and patient response to antidepressants. Depression symptoms might be associated with the duration of microstate B and anxiety symptoms related to the occurrence of microstate A. Therefore, the duration of microstate B and the occurrence of microstate A are potential biological markers for MDD patients' early response and further clinical outcomes.

## Introduction

Major depressive disorder is a prevalent psychiatric illness and one of the leading causes of disability across the world (1). Antidepressants are still the most commonly used treatment for depression. Although most patients respond to conventional antidepressants, the time to response is often 2 weeks or longer, which precludes doctors from determining whether to continue their treatment or make an adjustment timely. Previous studies suggest that patients' early improvement is highly sensitive in predicting subsequent treatment outcomes (2). As many studies showed that the improve of depression is related to the recovery of brain network function (3), it is crucial to identify predictive biomarkers for clinical efficacy at the early stage of treatment with antidepressants.

EEG, as a detection method with high temporal resolution, has attracted more and more attention due to its non-invasiveness and convenience. While many studies that used functional magnetic resonance imaging (fMRI) have shown that MDD is characterized by abnormal functional connections and neural activities (4), fMRI lacks the temporal resolution needed to track the dynamics of brain activities. Spontaneous activities of various large-scale cortical networks can be reflected in macroscopic EEG potentials (5, 6). The abnormality in this brain functional connectivity is associated with MDD (7), and can be affected by treatments. With the development of brain imaging technologies and computer algorithm measures, changes of brain networks have also been observed using EEG, which may help identify state biomarkers specific to MDD progression (8).

EEG microstates are canonical voltage topographies that reflect frequent activations of resting-state brain network components. According to numerous previous studies, EEG microstates are categorized into four canonical maps (i.e., class A, B, C, and D). Resting-state EEG topographies and the time series map configurations do not change randomly and continuously, but remain quasi-stable for a certain length of time and then change into a new map configuration that remains temporarily stable again (9, 10). One microstate class remains stable for around 80–120 ms before rapid transitioning to another microstate class (11). As a method with high test-retest reliability, resting-state microstates might be used to assess brain network activities (12). These results support the idea that the microstate may represent the status of information processing in a distributed neural network. Therefore, EEG microstates are potential candidates for “atoms of thought” (13).

The prevailing hypothesis states that EEG microstates become active with the changes of stimuli or circumstance, which has led to the development of microstate analysis, i.e., the identification and quantification of these topographies (14, 15). Advancements in clustering algorithms and computing power have allowed for a more detailed study of microstates. Many studies have confirmed the fundamental principle that a large quantity of the EEG signals can be represented by a small number of characteristic microstates (16, 17). Consistently, four canonical sets of microstates (i.e., A-D) have been identified in most studies (16). Since a microstate is relatively stable in a specific space and time range, this detection method requires high temporal resolution to obtain a particular state. To mediate complex mental activities and optimally respond to rapidly changing inputs, networks must be reorganized into different spatial patterns on a sub-second time scale (11, 18, 19). While microstate analysis has been widely used to study brain functions, few studies have investigated microstates in affective disorders (11). By comparing patients with MDD, bipolar depression, and healthy participants, some previous studies found that changes in resting-state EEG microstates in patients with depression were associated with brain network dynamics (20, 21).

To our knowledge, there have been few studies exploring affective disorders using EEG microstate analysis, and those cross-sectional studies could not longitudinally reflect the changes of EEG microstates in a certain period. To date, the micro-changes in MDD patients treated with antidepressants are yet to be reported. Therefore, we aim to identify predictive biological markers for patients' early response to treatment through exploring changes in EEG microstates in patients at the acute stage of MDD, in order to provide a clinical guidance for subsequent treatment for this disease.

## Methods

### Subjects

The study subjects were drug-naïve patients recruited through advertisement in the outpatient clinic of the Second Xiangya Hospital of Central South University. Fifty-three patients were screened, and 30 were enrolled and have completed the 2-week EEG recording (at baseline and 2-weeks of treatment) and clinical assessments (at baseline, 2-weeks and 3 months of treatment). All the subjects received medications according to their individualized prescription, and were on antidepressant monotherapy to avoid the impact of combination of drugs; their doses increased to an effective therapeutic dose within 1 week according to individual tolerance. All the subjects enrolled were drug-naïve adults who met the International Classification of Disease (ICD-10) criteria for MDD and had a baseline Hamilton Depression Rating Scale (HDRS-17) score of ≥17.

All the included subjects were assessed for depression and anxiety conditions using HDRS and HAMA (Hamilton Anxiety Rating Scale). Subjects were excluded if they were pregnant, had a significant risk for suicide, a history of neurological disorders, seizures, severe somatic diseases, or a history of drug or alcohol dependence or personality disorders. All the participants gave their written informed consent, and this study was approved by the Medical Ethics Committee of the Second Xiangya Hospital under the declaration of Helsinki and was registered on ClinicalTrials.gov (NCT04160377).

### EEG Recording

EEG was recorded at baseline and 2 weeks of treatment using an International 10–20 64-channel Ag-AgCl electrode EasyCap recording cap (http://easycap.brainproducts.com, EasyCap, Germany) while the subjects were sitting in a soundproof, shielded recording room. Data were collected at a 5,000 Hz sampling rate regarding the bilateral mastoid process. Electrode-impedances were kept below 5 kΩ. For the 10-min resting-state data, 5 min of EEG was recorded with eyes open and 5 min with eyes closed. Consistent with previous works, only data collected under an eyes-closed state were analyzed. The data were imported to BrainVision Analyzer 2.0 (Brain Products GmbH, Gilching, Germany), with epochs with eye movements, blinks, muscle activities, or other artifacts excluded. Artifactual segments were identified by visual inspection and removed, and eye blinks and electrocardiogram artifacts were removed using independent component analysis (ICA) (22, 23). Corrupted channels were visually identified and spline interpolated (24). Data were then re-referenced to the average reference.

### Microstate Analysis

Generally, microstate analysis is a powerful data-driven approach for the functional mapping of large-scale brain networks. The data were imported into MATLAB (The MathWorks. Inc.Natick, MA, USA) for processing. The open-source signal processing functions available in the EEGLAB toolbox version 13b were used for data importing and preprocessing (25). The continuous data were divided into 2-s segments, and a down notch filter (band-stop: 48–51 Hz) was used to remove the 50 Hz frequency further to minimize contamination of high-frequency artifacts. All the pieces were manually reviewed, and the trials and channels with any eye movements, muscle or body movements, or other non-physiological artifacts were discarded. The global field power (GFP) of each patient across 64 electrodes was calculated. For microstate segmentation, we down sampled the data to 250 Hz, had them band-pass filtered to 2–40 Hz, and included the time points containing the GFP (i.e., the local maximum) for analysis. Microstate transitions preferentially occur at GFP local minimal, which does not significantly impact the microstate segmentation results. The data were processed through a series of polarity-insensitive K-means clustering analyses ranging from 2 to 20 Hz. For microstate fitting, the microstate map that most closely resembled polarity was selected for each time point in the original data. A sequence of microstate labels with the same length was obtained as the original data set. For each record and microstate, four parameters, namely duration, occurrence, coverage, and transition, were calculated.

### Statistics

The subjects were assessed for severity of depression using HDRS at enrollment and after treatment, with a score decrease (reflecting clinical improvement) of at least 50% reflecting response to their antidepressant. Microstates were identified as continuous data for each subject, and all the topographies were assigned to the same class. The microstate duration (“duration”) refers to the average duration of a microstate class in milliseconds, occurrences per second represents the number of each microstate appearing per second (“occurrence”), and coverage means the percentage of each state occupying the total time analyzed (“coverage”). Paired *t*-test was performed to compare each class of microstate before and after treatment. The relationship between improvement in HAMA score after 2 weeks of treatment and the components of the microstate was examined using Spearman correlation coefficient. Linear correlation analysis was conducted between microstate changes and the change of HDRS scores. For all the above analyses, the significance level was set at *p* = 0.05.

## Results

### Clinical Measures

A total of 30 patients completed the 2-week treatment, and 3 failed to respond to their antidepressant, as their HDRS score reduction was <20%. Eleven subjects had an HDRS score reduction of more than 50% (*P* < 0.001). For the MDD patients, the lowest and highest scores of HAMA were nine and 28, respectively; the lowest and highest scores of HDRS were 17 and 36, respectively. Significant difference in HAMA and HDRS after 2 weeks treatment with antidepressant (see Table 1). Additional clinical information is reported in Supplementary Table 1.

**Table 1:** Demographic and clinical data.

	**Before treatment**	**After treatment**	**T, P**
Sample size	30		
Sex (male/female)	10/20		
Age (year), mean (SD)	23.90 (4.57)		
Education (year), mean (SD)	15.20 (2.58)		
Duration disease (month)	5.77 (3.40)		
HDRS	22.13 (4.77)	12.40 (4.66)	10.442, <0.001
HAMA	18.77 (5.74)	12.03 (5.28)	5.753, <0.001

### EEG Microstate Analysis

**Figure 1:**
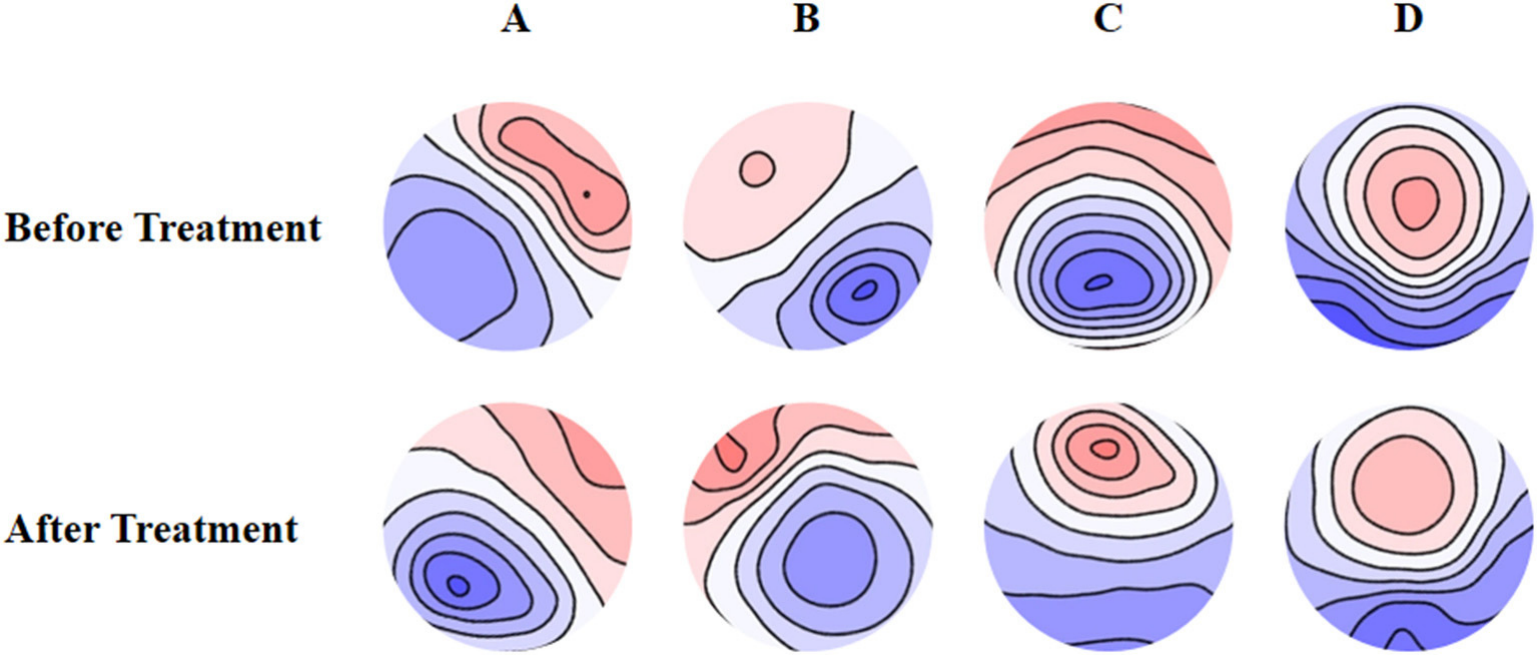
Spatial configuration of the four microstate classes. **(A)** Microstate A; **(B)** microstate B; **(C)** microstate C; **(D)** microstate D. Blue regions are negative and red are positive relative to average reference. The top row shows the EEG topoplots for the microstates identified before treatment, and the row under it shows the EEG topoplots for the microstates identified after treatment with anti-depressants.

The mean maps of the four microstate classes are shown in Figure 1. They closely resembled the classes found in previous EEG results with subjects' eyes closed and were labeled accordingly (9). Classes A and B were defined as oriented axes of the mapped field, class C as a clear anterior-posterior orientation, and class D as a fronto-central extreme location. The four microstate classes' explained variance was 75.8% (SD: 3.2%) before treatment and 75.6% (SD: 2.9%) after treatment.

Compared with the baseline, the mean duration of microstate B significantly decreased (*t* = 3.616, *p* = 0.001), the number of occurrences per second also significantly decreased (*t* = 3.364, *p* = 0.002), and the coverage of microstate B significantly decreased (*t* = 3.701, *p* = 0.001) after 2 weeks of treatment. We also found significant increase of the occurrence (*t* = −4.273, *p* < 0.001) and coverage (*t* = −3.457, *p* < 0.002) of microstate A. The changes in the remaining microstates were not significant (Table 2).

**Table 2:** Microstate parameters in patients with MDD (*n* = 30).

	**Before treatment**		**After treatment**		***t*-value**	***p*-value**
	**Mean**	**S.D**.	**Mean**	**S.D**.		
**Duration (ms)**						
Class A	66.00	11.92	69.67	14.02	−1.613	0.118
Class B	69.67	10.33	64.00	7.70	3.616	0.001
Class C	68.33	14.40	67.33	12.02	0.414	0.682
Class D	64.00	11.63	63.00	10.55	0.532	0.599
**Occurrence (times/s)**						
Class A	3.33	0.58	3.91	0.52	−4.273	<0.001
Class B	4.06	0.69	3.69	0.70	3.364	0.002
Class C	4.04	0.79	4.06	0.55	−0.182	0.857
Class D	3.82	0.78	3.81	0.70	0.107	0.915
**Coverage (%)**						
Class A	21.60	5.59	26.53	5.56	−3.457	0.002
Class B	27.53	5.81	23.23	4.61	3.701	0.001
Class C	27.13	7.18	26.67	4.81	0.333	0.742
Class D	23.60	5.82	23.67	4.92	−0.065	0.948

### Microstate Transitioning

**Figure 2:**
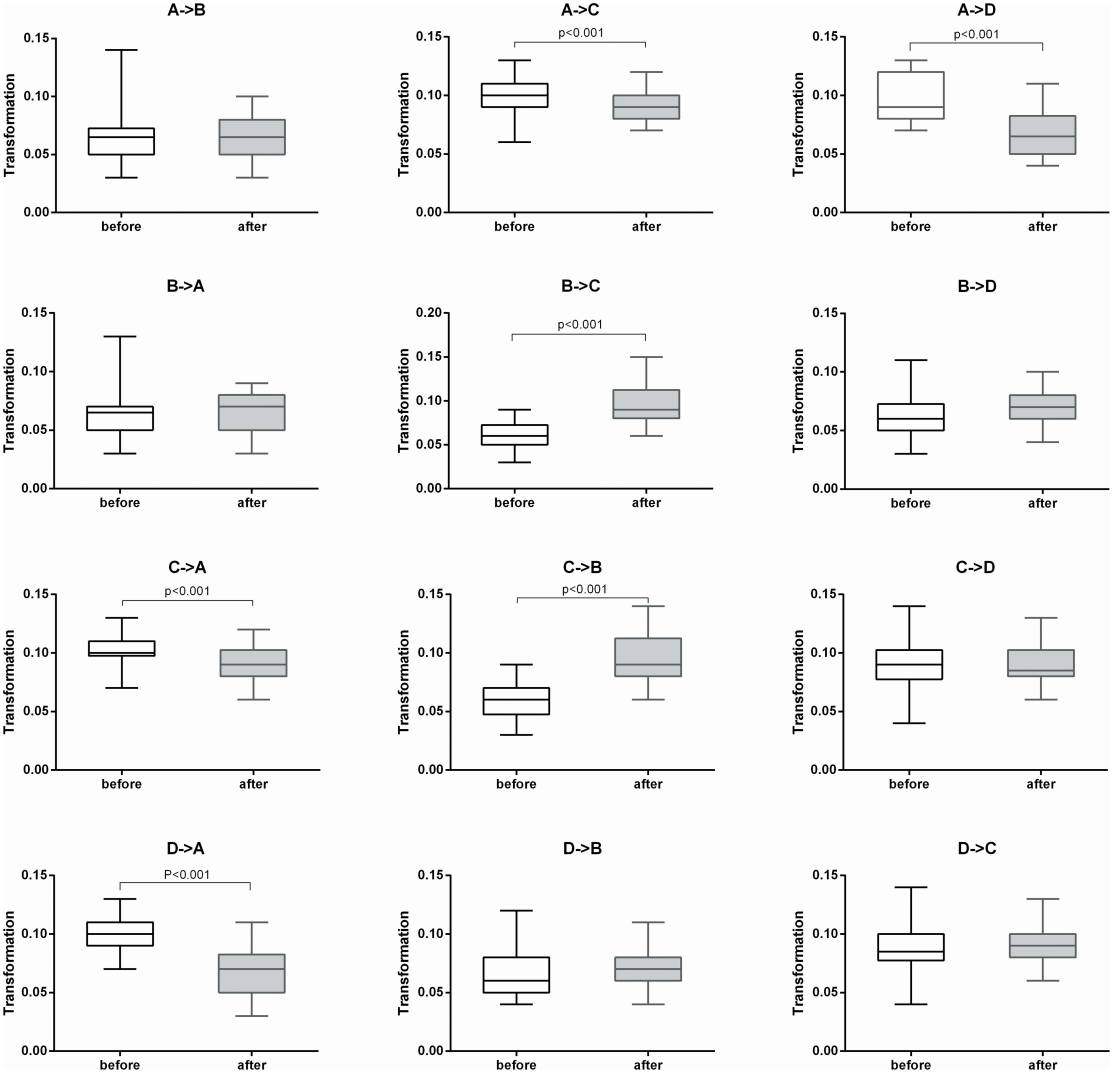
Microstate transitioning probabilities of the four microstate categories before and after treatment (*n* = 30).

After 2 weeks of treatment, the probability of transitioning from microstate A to C and D increased significantly (*t* = −4.114, *p* < 0.001 and *t* = −2.355, *p* < 0.05, respectively), while the probability of transitioning from microstate B to C and D decreased significantly (*t* = 3.579, *p* < 0.001 and *t* = 3.579, *p* < 0.001, respectively). The probability of transitioning from microstate C to A significantly increased (*p* < 0.001), while transitioning from C to B significantly decreased (*p* = 0.001). The probability of transitioning from microstate D to A significantly increased (*p* < 0.001). The probability of transitioning increased for microstates A ⇌ C and A ⇌ D, while significantly decreased for microstates B ⇌ C. The probability of transitioning of microstates A ⇌ B and C ⇌ D showed no significant changes (Figure 2).

### The Relationship Between EEG Microstate Parameters and Clinical Outcomes

**Figure 3:**
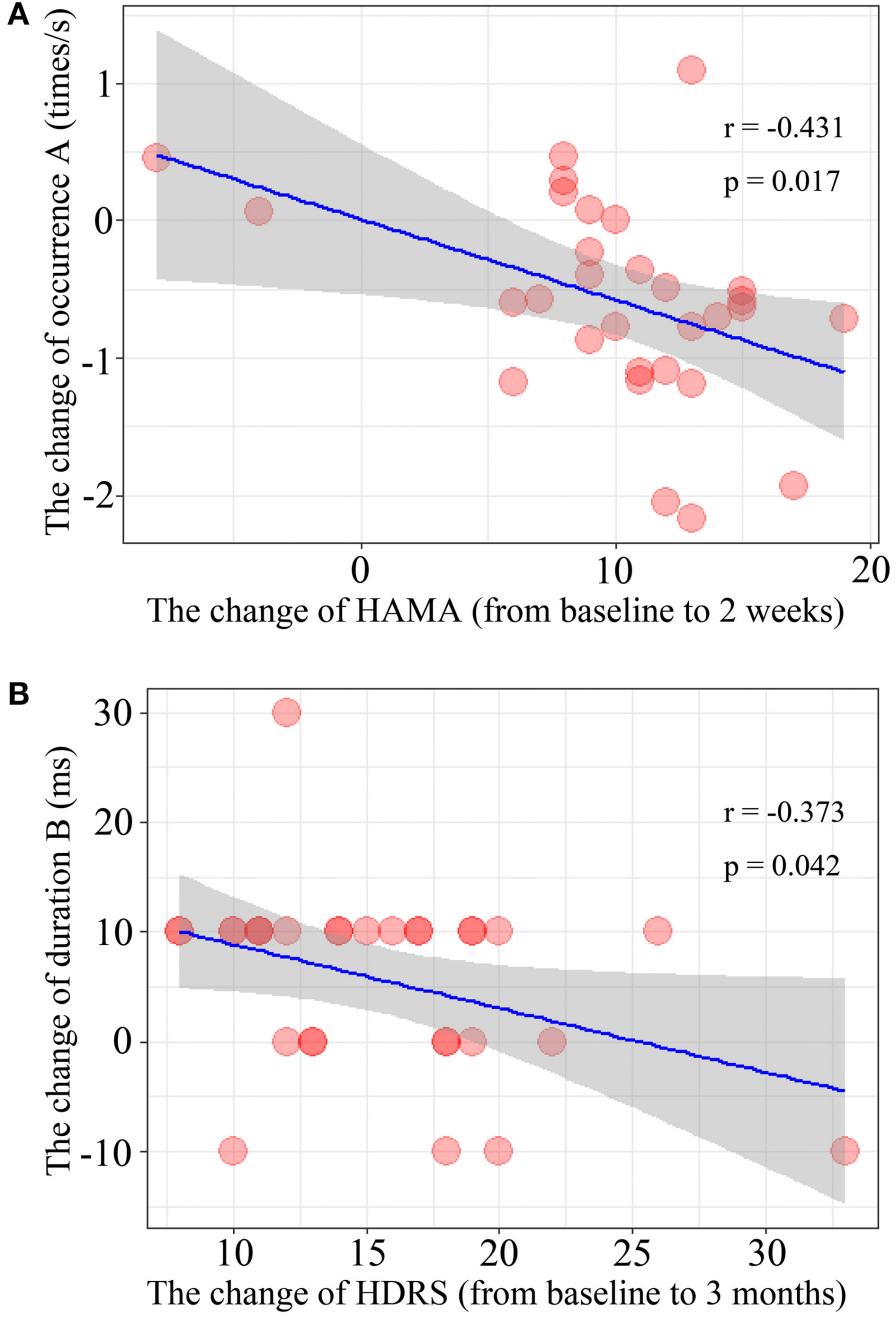
Correlation of the microstate parameters and clinical outcomes. **(A)** The changes of occurrence of microstate class A and the change of HAMA score after treatment 2 weeks. **(B)** The relationship between the change of duration of microstate class B during 2-weeks and the change of HDRS score after treatment 3 month (from baseline to 3-months).

The results of Spearman's rank correlation revealed a significant negative correlation between the reduction in HAMA score and the occurrence of microstate A (*r* = −0.431, *p* = 0.017) after 2 weeks of treatment. Moreover, the reduction of HDRS score after 3 months of treatment was also negatively correlated with the change in duration of microstate B (*r* = −0.373, *p* = 0.042) (Figure 3).

## Discussion

The aim of this study is to identify the EEG microstate parameters for patients' response to treatment with antidepressant. From the above three verifications, we concluded that the decrease of microstate class B is associated with the short-term clinical effect. We also found that the increase in occurrence of microstate A was negatively correlated with the reduction of HAMA score after 2 weeks of antidepressant treatment, indicating that increased occurrence of microstate A was associated with the improvement of anxiety symptoms. Therefore, the occurrence of microstate A might be an electrophysiological marker for early antidepressant efficacy. Increased occurrence of microstate A indicates increased alpha activity related to cognition in the anterior left prefrontal lobe. For the severity of depression, there was a significant negative correlation between the reduction of HDRS score at 3 months after treatment and the change in the duration of microstate B after 2 weeks of treatment. We also found that effective antidepressant treatment is associated with a shorter duration of microstate B. Previous studies reported that microstate B is associated with greater right posterior alpha activity (7, 17). Therefore, we consider that the duration of microstate B might be a stable physiological indicator for the effectiveness of antidepressant treatment, and changes in duration B during 2 weeks of treatment may predict patient response at 3 months of treatment.

The present results indicate that the patients with MDD had alterations of brain network dynamics at a sub-second level. This is consistent with the results of an fMRI study, which suggested that abnormalities in EEG microstates in patients with MDD and microstate probabilities of transitioning exhibited multiple significant differences, primarily in class A and C, compared with healthy controls and patients with remitted MDD (21). In the present study, the occurrence of microstate A significantly increased, and transition probabilities between A and C and between A and D also increased after 2 weeks of treatment, which is consistent with the results of previous studies on microstate transitioning. A previous study of Alzheimer's disease found that microstate A was associated with impaired cognitive function, suggesting that this microstate class might be an early biological marker for this disease (26). Furthermore, we found that the occurrence of microstate B significantly decreased, which was not reported in previous studies. Microstate class C was the most critical microstate class in healthy controls during resting state with eye closed (9). Studies showed that the duration of microstates was related to cognitive processing; for example, a study showed that attention deficits in MDD were associated with a shorter microstate duration, which indicated less sustained brain activities (27).

The present study also found that the probability of transitioning from microstate class C to B and from B to C significantly increased after treatment. The dynamics of microstates might also be related to the rapid reorganization and adaptation of brain networks (28). One possible explanation is that the decrease in the occurrence of microstate class C is associated with difficulty in the cognitive evaluation of circumstances. These results further support the idea that the frequency of brain information exchange significantly increased in depressed patients in the resting state. Therefore, we may infer that microstate C in patients with depression is consistent with the deactivation mode of the executive control network under fMRI (29). Indeed, the relationship between a microstate and cognitive functions are complex, and one cognitive process might involve multiple microstate classes (30).

## Limitations

There are several limitations in this study: (1) a relatively small number of subjects were included in the analyses, and the influence of gender might not be excluded; (2) no control group was included in this study; (3) investigation of the source location of EEG microstates requires higher spatial resolution, which cannot be provided using EEG only; (4) in addition to the four canonical classes of microstates described above, some other classes also need to be investigated, as they might have a potential influence on brain functions; (5) future research may require a long follow-up period to track the changes in EEG microstates in patients with MDD.

## Conclusions

In this study, we explored the characteristics of microstates before and after 2 weeks of treatment among drug-naïve patients with MDD using resting EEG recordings. Although the change of microstates might only be temporary, it might be a sensitive indicator for depressive symptoms and behavioral changes, as well as patients' response to antidepressants. Specifically, microstate B may be an essential parameter for the prediction of clinical efficacy. Also, microstate A was found to be related to the anxiety symptoms. Therefore, the EEG microstate is a sensitive and promising indicator for early prediction of patients' anxiety symptoms and response to antidepressants.

## Data Availability Statement

The raw data supporting the conclusions of this article will be made available by the authors, without undue reservation.

## Ethics Statement

The studies involving human participants were reviewed and approved by Medical Ethics Committee approved the Second Xiangya Hospital. The patients/participants provided their written informed consent to participate in this study.

## Author Contributions

LL, YZ, and ML conceived and designed the experiments. XL, SW, LZ, and DY performed the experiments. JL, BL, and WO analyzed the data. ZL and HL contributed materials/analysis tools. DY wrote the paper. All authors contributed to the article and approved the submitted version.

## Conflict of Interest

The authors declare that the research was conducted in the absence of any commercial or financial relationships that could be construed as a potential conflict of interest.

## Publisher's Note

All claims expressed in this article are solely those of the authors and do not necessarily represent those of their affiliated organizations, or those of the publisher, the editors and the reviewers. Any product that may be evaluated in this article, or claim that may be made by its manufacturer, is not guaranteed or endorsed by the publisher.
