# Supplementary material for: Prediction of Clinical Outcomes With EEG Microstate in Patients With Major Depressive Disorder
Source: Front Psychiatry. 2021 Aug 16;12:695272. doi: 10.3389/fpsyt.2021.695272 (PMC8415359; doi:10.3389/fpsyt.2021.695272)
Supplement: Supplementary file 1 [file Table_1.docx]

**Supplementary 1**

**Comparison of HAMA and HDRS scores before and after treatment [(x̅ ± SD), score]**

|  | Time | Scores |
| --- | --- | --- |
| HAMA (n=30) | Baseline | 18.77 ±5.74 |
|  | After 2 weeks | 12.03 ±5.28^a*^ |
|  | After 3 months | 11.53 ±4.49^b*^ |
| HDRS (n=30) | Baseline | 22.13 ±4.77 |
|  | After 2 weeks | 12.40 ±4.66^a*^ |
|  | After 3 months | 6.60 ±2.69^b*^ |

Note: a: Two weeks after treatment compared with baseline; b: Three months after treatment compared baseline; *P<0.001.

HAMA: Hamilton Anxiety Scale; HDRS: Hamilton Depression Rating Scale.
